# Diet-Related Inflammation Is Associated with Worse COVID-19 Outcomes in the UK Biobank Cohort

**DOI:** 10.3390/nu15040884

**Published:** 2023-02-09

**Authors:** Longgang Zhao, Michael D. Wirth, Fanny Petermann-Rocha, Solange Parra-Soto, John C. Mathers, Jill P. Pell, Frederick K. Ho, Carlos A. Celis-Morales, James R. Hébert

**Affiliations:** 1 Department of Epidemiology and Biostatistics, Arnold School of Public Health, University of South Carolina, Columbia, SC 29208, USA; 2 Cancer Prevention and Control Program and Department of Epidemiology and Biostatistics, University of South Carolina, Columbia, SC 29208, USA; 3 College of Nursing, University of South Carolina, Columbia, SC 29208, USA; 4 Department of Nutrition, Connecting Health Innovations LLC, Columbia, SC 29201, USA; 5 School of Cardiovascular and Medical Sciences, University of Glasgow, Glasgow G12 8TA, UK; 6 Centro de Investigación Biomédica, Facultad de Medicina, Universidad Diego Portales, Santiago 8370068, Chile; 7 Department of Nutrition and Public Health, Universidad del Bío-Bío, Chillan 3780000, Chile; 8 Human Nutrition & Exercise Research Centre, Centre for Healthier Lives, Population Health Sciences Institute, Newcastle University, Newcastle upon Tyne NE2 4HH, UK; 9 School of Health and Wellbeing, University of Glasgow, Glasgow G12 8RZ, UK; 10 Human Performance Lab, Education, Physical Activity and Health Research Unit, University Católica del Maule, Talca 3466706, Chile

**Keywords:** DII, COVID-19, cohort, nutrition, inflammation

## Abstract

Diet, the most important modulator of inflammatory and immune responses, may affect COVID-19 incidence and disease severity. Data from 196,154 members of the UK biobank had at least one 24 h dietary recall. COVID-19 outcomes were based on PCR testing, hospital admissions, and death certificates. Adjusted Poisson regression analyses were performed to estimate the risk ratios (RR) and their 95% confidence intervals (CI) for dietary inflammatory index (DII)/energy-adjusted DII (E-DII) scores. Models were adjusted for sociodemographic factors, comorbidities, smoking status, physical activity, and sleep duration. Between January 2020 and March 2021, there were 11,288 incident COVID-19 cases, 1270 COVID-19-related hospitalizations, and 315 COVID-19-related deaths. The fully adjusted model showed that participants in the highest (vs. lowest) DII/E-DII quintile were at 10–17% increased risk of COVID-19 (DII: RR _Q5 vs_. _Q1_ = 1.10, 95% CI 1.04–1.17, P_trend_ < 0.001; E-DII: RR _Q5 vs_. _Q1_ = 1.17, 95% CI 1.10–1.24, P_trend_ < 0.001) and ≈40% higher risk was observed for disease severity (DII: RR _Q5 vs_. _Q1_ = 1.40, 95% CI 1.18–1.67, P_trend_ < 0.001; E-DII: RR _Q5 vs_. _Q1_ = 1.39, 95% CI 1.16–1.66, P_trend_ < 0.001). There was a 43% increased risk of COVID-19-related death in the highest DII quintile (RR _Q5 vs_. _Q1_ = 1.43, 95% CI 1.01–2.01, P_trend_ = 0.04). About one-quarter of the observed positive associations between DII and COVID-19-related outcomes were mediated by body mass index (25.8% for incidence, 21.6% for severity, and 19.8% for death). Diet-associated inflammation increased the risk of COVID-19 infection, severe disease, and death.

## 1. Introduction

The COVID-19 pandemic continues to pose a threat to global public health. Relatively early in the pandemic, the European Society for Clinical Nutrition and Metabolism (ESPEN) issued expert statements and practical guidance for the nutritional management of individuals already diagnosed with SARS-CoV-2 infection, including those at high risk of needing intensive care [1]. Over a century of research has established that dietary factors are centrally important for mounting appropriate inflammatory and related immune responses against infectious disease agents, including viruses of all classes and enteropathogenic bacteria [2,3,4,5,6]. Therefore, the relative dearth of evidence about diet or nutrition concerning COVID-19 is a significant research gap.

To date, a limited number of studies have evaluated the role of specific food groups in the onset and progression of SARS-CoV-2 infection and COVID-19 symptoms [7,8,9]. However, because foods and nutrients are not eaten in isolation, over the last two decades, nutritional epidemiology has moved decisively to investigate links between patterns of dietary intake and health [10,11]. Consistent with this approach, Morais et al. suggested that a whole-diet approach could be effective in preventing COVID-19 [12]. However, they did not present any data to support their hypothesis. Recently, it was found that a healthy plant-based diet as indicated by better Alternative Healthy Eating Index (AHEI)-2010 or the Alternative Mediterranean Diet (AMED) scores [13] was associated prospectively with a lower risk of COVID-19 infection but not the severity of the disease.

As with many infectious diseases, COVID-19 is associated with the dysregulation of inflammatory response pathways [12,14]. While there is evidence that diet may be involved in the etiology of COVID-19 [12], most research on links between diet or nutrition and COVID-19 has focused on symptom management in those diagnosed with COVID-19 [14,15,16]. Comorbid conditions, including obesity, and cardio-metabolic diseases that increase the risk of COVID-19 [17,18] are also associated with worse prognosis [1,19,20,21]. In particular, a recent systematic review and meta-analysis showed strong relationships between higher body mass index (BMI) and risk of hospitalization, admissions to the intensive care unit, requiring invasive mechanical ventilation, and death from COVID-19 infection [22]. Comorbid metabolic conditions are also associated with inflammation, both as cause and sequela [23,24,25,26]. Previous work showed that the Dietary Inflammatory Index (DII^®^), which was created to quantify the effect of diet on inflammation [27,28], is related to gastrointestinal symptoms associated with infection [29]. Additionally, DII scores have been associated with airway inflammation [30]. Although the hypothesis is enticing [31], there is little evidence about associations between DII and respiratory infection.

This study aimed to investigate associations between dietary inflammatory potential and the risk of COVID-19 using a large population-based cohort, the UK Biobank. We hypothesized that a higher DII score, indicating a more pro-inflammatory diet, is associated with (1) increased risk of COVID-19, (2) increased likelihood of more severe disease, and (3) increased likelihood of COVID-19-related death. In addition, we hypothesized that these associations with DII were partially mediated by adiposity.

## 2. Materials and Methods

### 2.1. Population

The current study was conducted using data from UK Biobank, a population-based prospective cohort of more than 500,000 participants, recruited between 2006 and 2010, aged 37–73 years old, who attended one of 22 assessment centers across the UK. During these baseline assessments, participants completed touch-screen questionnaires, and physical measurements were performed. Details about the UK Biobank can be found elsewhere [32].

A modified 24 h dietary recall, the Oxford WebQ, was used to collect dietary information in the later stages of the baseline survey and during early follow-up. Therefore, for the current analyses, we restricted our study population to individuals who completed at least one reliable WebQ (*n* = 211,002). The proportions of participants who completed one, two, three, four, or five 24 h dietary assessments were 40% (*n* = 84,031), 23% (*n* = 48,260), 20% (*n* = 42,302), 14% (*n* = 29,928), and 3% (*n* = 5605), respectively [33]. After the exclusion of participants who died before the beginning of the COVID-19 pandemic on 13 January 2020 (*n* = 7661), had extreme estimates of energy intake (*n* = 4135), or had missing values in important covariates (*n* = 3052), the total analytic sample size was 196,154 (Supplemental Figure S1).

### 2.2. Exposures

The Oxford WebQ, which queried 206 foods and 32 beverages consumed during the past 24 h, was used to collect dietary information. Nutrient intake was calculated using McCance and Widdowson’s Composition of Foods [34]. Results from a validation study showed the mean Spearman correlation coefficient between the Oxford WebQ and the Interviewer-administered 24 h dietary recall for 21 nutrients was ≈0.6 with the majority ranging from 0.5 to 0.9 [35].

DII scores were calculated based on the mean of the available Oxford WebQ dietary data using methodology described elsewhere. To account for differences in total caloric intake, the energy-adjusted DII (E-DII^TM^) was also created. The rationale and description of E-DII methodology were published in 2019 [27,28]. In about ⅔ of studies, the E-DII has better predictive ability compared with DII. A total of 18 of the possible 45 food parameters were used for DII calculation in the UK Biobank and data on 17 food parameters for calculation of E-DII (as energy was in the denominator).

### 2.3. Outcomes

The primary outcomes of our study were severe COVID-19 infection, defined as hospitalization for COVID-19, and mortality due to COVID-19 infection. COVID-19-related hospitalizations and deaths were defined as ICD-10 code U07.1 or U07.2. Data on hospital admissions were obtained from Hospital Episode Statistics (HES; England) and Scottish Morbidity Records (Scotland) and were available up to 31 March 2021. Follow-up was censored on this date. COVID-19-related deaths were collected through certified death records and were available until 28 February 2021. Follow-up was censored on this date or date of death, from any cause, if this occurred earlier. COVID-19 PCR test results were available from 13 January 2020. We further considered confirmed COVID-19 infection, defined as having at least one positive PCR test result, as our secondary outcome.

### 2.4. Covariates

Extensive information on sociodemographic status, lifestyle, general health, and medical history was self-reported using a touch-screen questionnaire. We selected the following variables as potential confounders *a priori*: age at baseline, sex (male, female), race/ethnicity (White, non-White), smoking status (never, previous, or current smoker), Townsend Deprivation Index [36], physical activity, and self-reported history of heart disease, cancer, and diabetes, sleep duration (<7 h, 7–9 h, >9 h). Race/ethnicity and smoking status were self-reported. Townsend Deprivation Index—a marker of area-based deprivation based on the postcode of residence—was derived from aggregated data on unemployment, car and home ownership, and household overcrowding—and was further categorized into population tertiles. BMI was further classified into three groups: normal weight (<25 kg/m^2^), overweight (25–29.9 kg/m^2^), and obese (≥30 kg/m^2^). Physical activity was self-reported using the validated International Physical Activity Questionnaire estimating the total metabolic equivalent of task (MET) per week and categorized into inactive, moderately active, active, and missing.

### 2.5. Patients’ Involvement

No patients were involved in the concept of the research question or the outcome measures. They were not involved in the design or implementation of the study. No patients were asked to share suggestions on interpreting or drafting the results.

### 2.6. Statistical Analyses

We described the population characteristics by quintiles of DII and E-DII. Median (interquartile) was used for continuous variables and frequency (percentage) for categorical variables. *p*-values were derived from the analysis of variance (ANOVA) test for continuous variables and Chi-square tests for categorical variables. For all analyses, DII and E-DII scores were treated as both continuous variables and as quintiles, in separate models. Poisson regression analyses were performed to estimate the risk ratio (RR) and its 95% confidence interval (CI) for COVID-19-related outcomes. Four models were conducted: (1) age- and sex-adjusted; (2) additionally adjusted for race/ethnicity, Townsend Deprivation Index; (3) additionally adjusted for disease history including heart diseases, cancer, and type 2 diabetes; and (4) additionally adjusted for other modifiable risk factors including smoking status, physical activity, and sleep duration. Tests for trends across ordered categorical variables were calculated using the median value for each category as a continuous variable in the Poisson regression model. We further performed stratified analyses to explore whether sex (male, female), race/ethnicity (Whites, non-Whites), history of heart diseases (yes, no), cancer (yes, no), type 2 diabetes (yes, no), smoking status (smoker, nonsmokers), sleep duration (<7 h, ≥7 h), BMI category (<25 kg/m^2^, ≥25 kg/m^2^), modified the association between DII/E-DII score and COVID-19-related outcomes. Interaction tests were performed by adding a product of the test variables and exposure to our multivariable-adjusted model. We further used restricted cubic spline regression with 4 knots (*a priori* selected at 0.05, 0.35, 0.65, 0.95) to test for nonlinear associations between DII/E-DII score and COVID-19. The *p*-value for nonlinearity was calculated by the Wald test to evaluate whether the coefficients of all spline terms equal zero. To assess the potential mediation of obesity, we performed causal mediation analyses treating both DII/E-DII score and BMI as continuous variables. We used the SAS procedure CAUSALMED to estimate the total effect, natural direct effect, natural indirect effect, and proportion mediated [37].

Several sensitivity analyses were conducted to test the robustness of our main results. We reanalyzed the associations between DII/E-DII and COVID-19 outcomes restricting the study sample to participants with at least one COVID-19 report (*n* = 68,820), and participants with non-missing values in physical activity (*n* = 167,013). We also re-ran the model for COVID-19 infection, using a wider definition of positive PCR test, as well as outcomes based on hospitalization for COVID-19 or death from COVID-19. For all tests, we used a two-sided *p*-value of 0.05 to assess significance. All statistical analyses were performed using SAS 9.4.

## 3. Results

### 3.1. Participant Characteristics

A total of 11,288 (5.8%) participants with at least one positive result in the COVID-19 PCR test were recorded among 196,154 participants who had dietary data needed to compute DII/E-DII scores. We documented 1270 severe COVID-19 cases and 315 deaths due to COVID-19. DII scores ranged from −4.27 to 3.35, with a median of −0.43 (IQR 1.72), and E-DII scores ranged from −4.93 to 3.33, with a median of −0.51 (IQR 2.00). Table 1 shows the baseline characteristics of the study population according to quintiles of DII and E-DII. Compared with participants in the lowest quintile of DII and E-DII, those in the highest quintile were younger, had higher BMI, and had higher Townsend scores. They were also more likely to be current smokers, less physically active, and more likely to have a history of cancer.

### 3.2. Association between Dietary Inflammatory Index and Risk of COVID-19

Table 2 shows the associations between DII/E-DII scores and COVID-19 outcomes. Both the DII and E-DII were significantly associated with confirmed COVID-19 infection and severe COVID-19 (both P_trend_ < 0.001). Compared with the lowest quintile of DII, the fully adjusted (Model 4) RRs (95% CI) for COVID-19 infection in the highest quintile of DII was 1.10 (1.04–1.17) with P_trend_ < 0.001. A stronger association was observed between E-DII and COVID-19 infection (RR _Q5 vs. Q1_ =1.17, 95% CI = 1.10–1.24, P_trend_ < 0.001). For severe COVID-19, the associations were even stronger; about 40% higher risk for those in quintile 5 compared with quintile 1 of DII or E-DII (DII: RR _Q5 vs. Q1_ = 1.40, 95% CI 1.18–1.67, P_trend_ < 0.001; E-DII: RR _Q5 vs_. _Q1_ = 1.39, 95% CI 1.16–1.66, P_trend_ < 0.001). In contrast, whilst DII and E-DII were positively associated with COVID-19-related death in the crude model, the association with E-DII was not significant after adjustment for potential confounders. We detected a nonlinear relationship between DII and COVID-19 incidence (P_nonlinearity_ = 0.03). The risk for COVID-19 incidence started to increase from the 3rd DII quintile and then increased consistently in the higher quintiles (Supplemental Figure S2).

In the stratified analyses, the observed positive associations between DII/E-DII score and COVID-19 incidence and severity were robust across sex, race/ethnicity, history of heart diseases, cancer, type 2 diabetes, smoking status, sleep duration, and overweight (Supplemental Figures S3 and S4). We observed a potential interaction between smoking status and DII/E-DII on the risk of COVID-19 incidence (P_interaction_ < 0.05). The positive associations between DII/E-DII and the risk of COVID-19 incidence were stronger among never smokers.

### 3.3. Mediation Analyses

We further evaluated the role of BMI on the associations between DII/E-DII scores and COVID-19-related outcomes (Table 3). For DII, the proportion mediated by BMI was 25.8% (95% CI: 11.9–39.8), 21.6% (95% CI: 12.2–31.1), and 19.8% (95% CI: 3.1–36.5), for COVID-19 incidence, severity, and death, respectively. The mediation effects between E-DII and COVID-19-related outcomes were similar to those based on DII.

### 3.4. Sensitivity Analyses

To examine the impact of missing results for COVID-19 testing, we reanalyzed our data for participants with at least one COVID-19 test. The results were similar to our main findings (Supplemental Table S1). Using a broader definition of COVID-19 infection produced no material changes to our main findings (Supplemental Table S1). We also found similar results among participants without missing values for physical activity (Supplemental Table S1).

## 4. Discussion

In this large prospective cohort, we found a consistent increase in the risk of confirmed COVID-19 infection and severe COVID-19 infection with increased DII and E-DII scores. These positive associations were mediated partially by obesity. The non-significant association with COVID-19 mortality, following adjustment, likely reflects lower statistical power due to many fewer endpoints (i.e., only 2.7% of all participants had at least one positive PCR test result and < 0.2% of the entire cohort of 196,154 eligible participants died of COVID-19).

Recently, several studies have evaluated the associations between diet quality and COVID-19 risk. One prospective cohort study based on the Nurses’ Health Study II and Health Professionals Follow-up Study found that better dietary quality, assessed as higher AHEI-2010 or AMED scores and lower Empirical Dietary Inflammatory Pattern (EDIP—developed in a population similar to the Nurses’ Health Study II and Health Professionals Follow-up Study) score, was associated with a lower likelihood of SARS-CoV-2 infection (OR _Q4 vs. Q1_ = 0.80, 0.78, and 1.13 for AHEI, AMED, and EDIP, all *p* for trend ≤ 0.01) [13]. Another, smartphone-based, COVID-19 Symptom Study involving 592,571 participants reported that a dietary pattern characterized by healthy plant-based foods was associated with lower risk and severity of COVID-19 (HR _Q4 vs_. _Q1_ = 0.91 and 0.59 for incidence and severity, respectively) [38]. Two case-control studies, conducted in Lebanon and Italy, indicated that higher adherence to the Mediterranean dietary pattern was associated inversely with SARS-CoV-2 infection [39,40].

The DII is predicated on the fact that dietary factors provide the substrate for the cytokine signaling necessary to regulate inflammatory and immune responses [28,41]. In addition to direct effects on inflammatory and immune responses, diets contain bioactive compounds that can divert substrates into metabolically unique pathways that produce distinct effects on inflammation [42]. For example, omega-3 fatty acids shunt metabolism away from the more pro-inflammatory cyclooxygenase pathway and toward the more anti-inflammatory lipoxygenase pathway [43]. They also produce resolvins, protectins, and maresins, which are needed for the negative feedback signaling necessary to resolve the acute inflammatory and innate immune response, which is necessary both to address the threat (in this case, SARS co-V2 infection) and to perform the reconnaissance necessary to stimulate the adaptive immune response necessary to prevent future damage [44]. They also prevent the organism from entering a state of chronic inflammation in which mounting another effective inflammatory/innate immune response is virtually impossible [45].

Individuals with comorbidities related to metabolic dysfunction [46] are typically in a state of chronic systemic or tissue-specific simmering inflammation that renders them less able to mount a competent immune response to infectious agents such as SARS co-V2 [47]. Results from this study are consistent both with the protective effects of an anti-inflammatory diet (which allows for a competent acute proinflammatory response) and the chronic proinflammatory effect of high levels of adiposity; here estimated by BMI [48].

To our knowledge, this investigation, based on the UK Biobank prospective cohort, is the largest study to examine the association between the DII/E-DII and COVID-19 outcomes. An earlier small case-control study, with 60 cases and 60 controls, reported that people diagnosed with COVID-19 were significantly more likely to be in the upper tertile of E-DII, compared with the bottom tertile (odds ratio = 11.86, 95% CI = 5.38–18.74) [49]. The retrospective design of that study makes it vulnerable to both survival and recall/information bias. Our study was strengthened by its prospective design, the large sample size, and full adjustment for important covariates.

Another strength of this study is its reliance on the DII/E-DII whose advantages over other dietary indices include that it (1) is grounded in peer-reviewed literature focusing specifically on inflammation; (2) can be adapted to virtually any dietary assessment method that provides estimates of nutrient intake; and (3) is standardized to dietary intake from representative populations around the world, thus facilitating quantitative comparisons across studies. Adding to the confidence that we have in the scoring algorithm, it is important to note that the DII/E-DII has been construct-validated in 42 populations around the world using hs-CRP, IL-4, 6, 8, and 10, TNF-α, calprotectin, fibrinogen, homocysteine, interferon-γ, WNT signaling, and five components of the kynurenine pathway [50,51,52,53].

Despite its strengths, our study has several limitations. First, dietary information was collected using a modified 24 h diet recall that employed a limited food and beverage list. Only 18 of the possible 45 components were available for DII computation. As such, some strongly anti-inflammatory foods may have been missed, thus reducing the power to capture the inflammatory potential from the whole diet. However, the use of a repeated dietary recall (Oxford WebQ in UK Biobank) is a robust approach for capturing habitual diet. So, it is advantageous that 60% of our participants have at least two 24 h diet recalls, which reduces the influence of intra-individual variability [54]. Second, COVID-19 infection was defined as having at least one positive PCR test. Whilst this obviates misclassification of symptoms due to other conditions, it also means that people unable to access PCR testing may not have been diagnosed with COVID-19. Lack of access to testing was systematically more likely earlier in the pandemic and among people with milder infections treated in the community. However, our sensitivity analyses, excluding those without PCR test results, showed that our results were stable and robust. Third, the definition of race/ethnicity includes broad categories of traits associated with ethnicity such as cultural heritage, language, religious traditions, other customs, and geography; and race, which attempts to divide people into groups based on physical and biological characteristics. Finally, although we controlled for numerous potential confounders in our multivariable-adjusted model, we cannot exclude the possibility of unmeasured confounding in this prospective observational study.

## 5. Conclusions

In the UK Biobank prospective cohort individuals with higher dietary inflammatory potential, measured by DII and E-DII scores, had a higher risk of confirmed COVID-19 infection and severe COVID-19 infection requiring hospitalization. These associations were independent of confounders and partially mediated by BMI, which is a proxy for adiposity. Improvement in dietary patterns may be beneficial in reducing the risk of COVID-19 and similar infections.

## Figures and Tables

**Table 1:** Characteristics of study population in the UK Biobank (*n* = 196,154).

Variable	Quintile of DII				Quintile of Energy-Adjusted DII			
	Quintile 1	Quintile 3	Quintile 5	*p* Value	Quintile 1	Quintile 3	Quintile 5	*p* Value
Total numbers	39,230	39,231	39,231		39,230	39,231	39,231	
Age, years	58 (12)	57 (12)	55 (14)	<0.001	58 (12)	57 (12)	55 (14)	<0.001
Body mass index, kg/m^2^	25.8 (5.3)	26.2 (5.4)	26.7 (5.9)	<0.001	25.8 (5.4)	26.1 (5.4)	26.9 (5.8)	<0.001
DII	−2 (0.6)	−0.4 (0.3)	1.1 (0.6)	<0.001	−1.9 (1.2)	−0.4 (0.9)	0.9 (1.0)	<0.001
E-DII	−2.1 (1.2)	−0.5 (1.1)	1 (1.1)	<0.001	−2.4 (0.8)	−0.5 (0.4)	1.2 (0.7)	<0.001
Sex (female)	17,043 (43.4)	17,544 (44.7)	17,211 (43.9)	<0.001	12,185 (31.1)	17,760 (45.3)	21,743 (55.4)	<0.001
White (yes)	37,820 (96.4)	37,865 (96.5)	36,783 (93.8)	<0.001	37,485 (95.6)	37,719 (96.1)	37,359 (95.2)	<0.001
Townsend Deprivation Index				<0.001				<0.001
Lower	14,636 (37.3)	14,300 (36.5)	12,517 (31.9)		14,604 (37.2)	14,219 (36.2)	12,859 (32.8)	
Middle	13,677 (34.9)	13,820 (35.2)	13,193 (33.6)		13,543 (34.5)	13,734 (35.0)	13,225 (33.7)	
Higher	10,917 (27.8)	11,111 (28.3)	13,521 (34.5)		11,083 (28.3)	11,278 (28.7)	13,147 (33.5)	
Central obesity (yes)	10,278 (26.2)	11,500 (29.3)	13,228 (33.7)	<0.001	10,509 (26.8)	11,270 (28.7)	13,426 (34.2)	<0.001
Smoking status				<0.001				<0.001
Never smoker	22,707 (57.9)	22,630 (57.7)	21,594 (55.0)		23,172 (59.1)	22,764 (58.0)	21,182 (54.0)	
Previous smoker	14,458 (36.9)	13,920 (35.5)	13,050 (33.3)		14,134 (36.0)	13,826 (35.2)	13,350 (34.0)	
Current smoker	2065 (5.3)	2681 (6.8)	4587 (11.7)		1924 (4.9)	2641 (6.7)	4699 (12.0)	
Cancer diagnosis (yes)	3023 (7.7)	2827 (7.2)	2637 (6.7)	<0.001	3114 (7.9)	2905 (7.4)	2571 (6.6)	<0.001
Heart disease (yes)	1604 (4.1)	1445 (3.7)	1630 (4.2)	0.007	1598 (4.1)	1510 (3.8)	1627 (4.1)	0.05
Diabetes (yes)	1402 (3.6)	1374 (3.5)	1539 (3.9)	0.004	1425 (3.6)	1404 (3.6)	1523 (3.9)	0.10
Physical activity				<0.001				<0.001
Inactive	4385 (11.2)	6236 (15.9)	7607 (19.4)		4561 (11.6)	6165 (15.7)	7578 (19.3)	
Moderate	13,822 (35.2)	14,368 (36.6)	13,928 (35.5)		13,832 (35.3)	14,385 (36.7)	13,944 (35.5)	
Active	15,783 (40.2)	12,878 (32.8)	11,243 (28.7)		15,145 (38.6)	12,878 (32.8)	11,600 (29.6)	
Missing	5240 (13.4)	5749 (14.7)	6453 (16.4)		5692 (14.5)	5803 (14.8)	6109 (15.6)	
Sleep duration				<0.001				<0.001
<7 h	8791 (22.4)	8434 (21.5)	9801 (25)		8892 (22.7)	8606 (21.9)	9699 (24.7)	
7 to <9 h	30,017 (76.5)	30,401 (77.5)	28,834 (73.5)		29,896 (76.2)	30,184 (76.9)	28,979 (73.9)	
≥9 h	422 (1.1)	396 (1.0)	596 (1.5)		442 (1.1)	441 (1.1)	553 (1.4)	

Data are presented as *n* (%) for categorical variables and median (interquartile) for continuous variables.

**Table 2:** Associations between DII, E-DII, and COVID-19 incidence, severity, and death in the UK Biobank.

		Quintile of DII or E-DII, RR (95% CI)							
		Quintile 1	Quintile 2	Quintile 3	Quintile 4	Quintile 5	P for Trend	Per SD Increase	P_Nonlinearity_
DII	Median level	−2.03	−1.12	−0.43	0.23	1.07			
Incidence									
	No. of cases	2037	2064	2307	2357	2523			
	Model 1	1 (reference)	0.99 (0.93–1.05)	1.09 (1.03–1.15)	1.09 (1.03–1.16)	1.12 (1.06–1.18)	<0.001	1.04 (1.03–1.06)	0.03
	Model 2	1 (reference)	0.99 (0.93–1.05)	1.09 (1.03–1.15)	1.09 (1.03–1.15)	1.11 (1.04–1.17)	<0.001	1.04 (1.02–1.06)	0.03
	Model 3	1 (reference)	0.99 (0.93–1.05)	1.09 (1.03–1.15)	1.09 (1.03–1.15)	1.10 (1.04–1.17)	<0.001	1.04 (1.02–1.06)	0.03
	Model 4	1 (reference)	0.99 (0.94–1.05)	1.09 (1.03–1.15)	1.09 (1.02–1.15)	1.10 (1.04–1.17)	<0.001	1.04 (1.02–1.06)	0.03
Severity									
	No. of cases	211	232	241	273	313			
	Model 1	1 (reference)	1.11 (0.92–1.33)	1.16 (0.97–1.40)	1.34 (1.12–1.60)	1.58 (1.33–1.89)	<0.001	1.18 (1.11–1.25)	0.32
	Model 2	1 (reference)	1.12 (0.93–1.35)	1.17 (0.97–1.40)	1.33 (1.11–1.59)	1.52 (1.27–1.81)	<0.001	1.16 (1.09–1.22)	0.64
	Model 3	1 (reference)	1.12 (0.93–1.35)	1.17 (0.97–1.41)	1.32 (1.10–1.58)	1.49 (1.25–1.78)	<0.001	1.15 (1.09–1.22)	0.73
	Model 4	1 (reference)	1.11 (0.92–1.34)	1.15 (0.95–1.38)	1.27 (1.06–1.53)	1.40 (1.18–1.67)	<0.001	1.12 (1.06–1.19)	0.84
Death									
	No. of cases	57	56	62	61	79			
	Model 1	1 (reference)	1.01 (0.70–1.45)	1.14 (0.80–1.64)	1.16 (0.81–1.67)	1.63 (1.16–2.29)	0.005	1.19 (1.05–1.34)	0.15
	Model 2	1 (reference)	1.02 (0.70–1.47)	1.14 (0.80–1.64)	1.16 (0.81–1.66)	1.56 (1.11–2.19)	0.01	1.17 (1.04–1.31)	0.27
	Model 3	1 (reference)	1.02 (0.70–1.47)	1.15 (0.80–1.65)	1.15 (0.80–1.64)	1.53 (1.09–2.15)	0.01	1.16 (1.03–1.30)	0.30
	Model 4	1 (reference)	1.01 (0.70–1.46)	1.13 (0.79–1.61)	1.10 (0.77–1.58)	1.43 (1.01–2.01)	0.04	1.13 (1.00–1.27)	0.37
E-DII	Median level	−2.4	−1.31	−0.51	0.25	1.22			
Incidence									
	No. of cases	1968	2119	2146	2444	2611			
	Model 1	1 (reference)	1.05 (0.99–1.11)	1.04 (0.98–1.10)	1.14 (1.08–1.21)	1.17 (1.11–1.24)	<0.001	1.05 (1.03–1.07)	0.17
	Model 2	1 (reference)	1.05 (0.99–1.11)	1.04 (0.98–1.10)	1.14 (1.08–1.21)	1.17 (1.10–1.23)	<0.001	1.05 (1.03–1.06)	0.20
	Model 3	1 (reference)	1.05 (0.99–1.12)	1.04 (0.98–1.10)	1.15 (1.08–1.22)	1.17 (1.10–1.24)	<0.001	1.05 (1.03–1.06)	0.22
	Model 4	1 (reference)	1.05 (0.99–1.12)	1.04 (0.98–1.10)	1.15 (1.08–1.21)	1.17 (1.10–1.24)	<0.001	1.05 (1.03–1.06)	0.26
Severity									
	No. of cases	204	231	228	283	324			
	Model 1	1 (reference)	1.09 (0.90–1.32)	1.06 (0.87–1.27)	1.30 (1.08–1.56)	1.48 (1.24–1.77)	<0.001	1.13 (1.08–1.19)	0.11
	Model 2	1 (reference)	1.10 (0.91–1.33)	1.06 (0.88–1.29)	1.31 (1.10–1.57)	1.46 (1.22–1.75)	<0.001	1.13 (1.07–1.18)	0.25
	Model 3	1 (reference)	1.11 (0.92–1.34)	1.08 (0.90–1.31)	1.34 (1.12–1.60)	1.48 (1.24–1.76)	<0.001	1.13 (1.07–1.19)	0.31
	Model 4	1 (reference)	1.11 (0.92–1.33)	1.06 (0.88–1.28)	1.29 (1.08–1.55)	1.39 (1.16–1.66)	<0.001	1.11 (1.05–1.16)	0.42
Death									
	No. of cases	53	68	58	59	77			
	Model 1	1 (reference)	1.22 (0.85–1.75)	1.03 (0.71–1.50)	1.05 (0.73–1.53)	1.40 (0.98–2.01)	0.17	1.10 (1.00–1.23)	0.15
	Model 2	1 (reference)	1.24 (0.87–1.78)	1.04 (0.72–1.52)	1.07 (0.74–1.56)	1.40 (0.98–1.99)	0.17	1.10 (0.99–1.22)	0.23
	Model 3	1 (reference)	1.26 (0.88–1.80)	1.06 (0.73–1.54)	1.10 (0.75–1.59)	1.41 (0.99–2.02)	0.14	1.11 (1.00–1.22)	0.26
	Model 4	1 (reference)	1.25 (0.87–1.79)	1.04 (0.72–1.51)	1.06 (0.73–1.54)	1.32 (0.92–1.89)	0.30	1.08 (0.98–1.20)	0.30

Abbreviations: CI, confidence intervals; DII, dietary inflammatory index; E-DII, energy-adjusted dietary inflammatory index; RR, risk ratio. Model 1 adjusted for age and sex. Model 2 additionally adjusted for race/ethnicity, and deprivation index. Model 3 additionally adjusted for history of heart disease, cancer, and type 2 diabetes. Model 4 additionally adjusted for smoking status, physical activity, and sleep duration.

**Table 3:** Detailed statistics of causal mediation analyses of body mass index on associations between DII/E-DII and COVID-19 outcomes.

	Effects	Incidence	Severity	Death
DII				
	Total effect	1.030 (1.014–1.047)	1.113 (1.061–1.166)	1.119 (1.013–1.225)
	Natural Direct effect	1.023 (1.006–1.039)	1.089 (1.037–1.140)	1.095 (0.991–1.199)
	Natural Indirect effect	1.008 (1.007–1.009)	1.022 (1.019–1.026)	1.021 (1.016–1.027)
	Proportion mediated (%)	25.82 (11.86–39.79)	21.64 (12.16–31.12)	19.77 (3.05–36.48)
E-DII				
	Total effect	1.039 (1.024–1.053)	1.094 (1.049–1.139)	1.074 (0.984–1.163)
	Natural Direct effect	1.032 (1.017–1.046)	1.073 (1.028–1.117)	1.053 (0.966–1.141)
	Natural Indirect effect	1.007 (1.006–1.008)	1.020 (1.017–1.023)	1.019 (1.014–1.024)
	Proportion mediated (%)	18.13 (11.06–25.19)	22.81 (12.30–33.32)	27.45 (−4.56 to 59.47)

BMI, body mass index. DII, dietary inflammatory index; E-DII, energy-adjusted dietary inflammatory index. Results were shown as risk ratio (95% confidence intervals) if not specified. The interaction between DII/E-DII and BMI is not significant in the above models. DII/E-DII and BMI were treated as continuous variables in the causal mediation analyses. Model adjusted for age, sex, ethnicity, deprivation index, smoking status, history of heart diseases, cancer, and type 2 diabetes, physical activity, and sleep duration.

## Data Availability

The full UK Biobank dataset is publicly available. The corresponding author should be contacted for code used in statistical analyses.

## Supplementary Materials

The following supporting information can be downloaded at: https://www.mdpi.com/article/10.3390/nu15040884/s1, Figure S1: Study selection procedure; Figure S2: Nonlinearity plots for associations between DII and COVID-19 incidence, severity, and death in the UK Biobank. Figure S3: Stratified analyses of associations between DII and COVID-19 incidence, severity, and death in the UK Biobank. Figure S4: Stratified analyses of associations between E-DII and COVID-19 incidence, severity, and death in the UK Biobank. Table S1: Sensitivity analyses on associations between DII, E-DII, and COVID-19 incidence, severity, and death in the UK Biobank.

## References

[1] Barazzoni R., Bischoff S.C., Breda J., Wickramasinghe K., Krznaric Z., Nitzan D., Pirlich M., Singer P. (2020). ESPEN expert statements and practical guidance for nutritional management of individuals with SARS-CoV-2 infection. Clin. Nutr..

[2] Scrimshaw N.S. (2010). INCAP Studies of Nutrition and Infection. Food Nutr. Bull..

[3] Chandra R.K. (1983). Nutrition, Immunity, and Infection: Present Knowledge and Future Directions. Lancet.

[4] Keusch G.T. (2003). The History of Nutrition: Malnutrition, Infection and Immunity. J. Nutr..

[5] Yap Y.-A., Mariño E. (2018). An Insight into the Intestinal Web of Mucosal Immunity, Microbiota, and Diet in Inflammation. Front. Immunol..

[6] Zinder R., Cooley R., Vlad L.G., Molnar J.A. (2019). Vitamin A and Wound Healing. Nutr. Clin. Pract..

[7] Deschasaux-Tanguy M., Srour B., Bourhis L., Arnault N., Druesne-Pecollo N., Esseddik Y., de Edelenyi F.S., Allègre J., Allès B., Andreeva V.A. (2021). Nutritional risk factors for SARS-CoV-2 infection: A prospective study within the NutriNet-Santé cohort. BMC Med..

[8] Holt H., Talaei M., Greenig M., Zenner D., Symons J., Relton C., Young K.S., Davies M.R., Thompson K.N., Ashman J. (2022). Risk factors for developing COVID-19: A population-based longitudinal study (COVIDENCE UK). Thorax.

[9] Zhou L., Li H., Zhang S., Yang H., Ma Y., Wang Y. (2022). Impact of ultra-processed food intake on the risk of COVID-19: A prospective cohort study. Eur. J. Nutr..

[10] Hu F.B. (2002). Dietary pattern analysis: A new direction in nutritional epidemiology. Curr. Opin. Lipidol..

[11] Steck S.E., Murphy E.A. (2020). Dietary patterns and cancer risk. Nat. Rev. Cancer.

[12] Morais A.H.D.A., Aquino J.D.S., da Silva-Maia J.K., Vale S.H.D.L., Maciel B.L.L., Passos T.S. (2021). Nutritional status, diet and viral respiratory infections: Perspectives for severe acute respiratory syndrome coronavirus 2. Br. J. Nutr..

[13] Yue Y., Ma W., Accorsi E.K., Ding M., Hu F., Willett W.C., Chan A.T., Sun Q., Edwards J.R., Smith-Warner S.A. (2022). Long-term diet and risk of SARS -CoV-2 infection and Coronavirus Disease 2019 (COVID-19) severity. Am. J. Clin. Nutr..

[14] Liu H., Zhou L., Wang H., Wang X., Qu G., Cai J., Zhang H. (2021). Malnutrition is associated with hyperinflammation and immunosuppression in COVID-19 patients: A prospective observational study. Nutr. Clin. Pract..

[15] Khan E. (2021). Heart failure and COVID-19: Synergism of two inflammatory conditions?. Br. J. Community Nurs..

[16] Sukkar S.G., Bassetti M. (2020). Induction of ketosis as a potential therapeutic option to limit hyperglycemia and prevent cytokine storm in COVID-19. Nutrition.

[17] Seclén S.N., Nunez-Robles E., Yovera-Aldana M., Arias-Chumpitaz A. (2020). Incidence of COVID-19 infection and prevalence of diabetes, obesity and hypertension according to altitude in Peruvian population. Diabetes Res. Clin. Pract..

[18] Fisman D.N., Tuite A.R. (2021). Evaluation of the relative virulence of novel SARS-CoV-2 variants: A retrospective cohort study in Ontario, Canada. Can. Med. Assoc. J..

[19] Fernández-Ayala D.J.M., Navas P., López-Lluch G. (2020). Age-related mitochondrial dysfunction as a key factor in COVID-19 disease. Exp. Gerontol..

[20] Mittal J., Ghosh A., Bhatt S.P., Anoop S., Ansari I.A., Misra A. (2021). High prevalence of post COVID-19 fatigue in patients with type 2 diabetes: A case-control study. Diabetes Metab. Syndr. Clin. Res. Rev..

[21] Allard L., Ouedraogo E., Molleville J., Bihan H., Giroux-Leprieur B., Sutton A., Baudry C., Josse C., Didier M., Deutsch D. (2020). Malnutrition: Percentage and Association with Prognosis in Patients Hospitalized for Coronavirus Disease 2019. Nutrients.

[22] Sawadogo W., Tsegaye M., Gizaw A., Adera T. (2022). Overweight and obesity as risk factors for COVID-19-associated hospitalisations and death: Systematic review and meta-analysis. BMJ Nutr. Prev. Health.

[23] Yi Q., Li X., He Y., Xia W., Shao J., Ye Z., Song P. (2021). Associations of dietary inflammatory index with metabolic syndrome and its components: A systematic review and meta-analysis. Public Health Nutr..

[24] Fu W., Pei H., Shivappa N., Hebert J.R., Luo T., Tian T., Alimu D., Zhang Z., Dai J. (2021). Association between Dietary Inflammatory Index and Type 2 diabetes mellitus in Xinjiang Uyghur autonomous region, China. PeerJ.

[25] Hariharan R., Odjidja E.N., Scott D., Shivappa N., Hébert J.R., Hodge A., de Courten B. (2022). The dietary inflammatory index, obesity, type 2 diabetes, and cardiovascular risk factors and diseases. Obes. Rev..

[26] Liao C., Gao W., Cao W., Lv J., Yu C., Wang S., Pang Z., Cong L., Wang H., Wu X. (2021). Associations of Metabolic/Obesity Phenotypes with Insulin Resistance and C-Reactive Protein: Results from the CNTR Study. Diabetes Metab. Syndr. Obes..

[27] Hébert J.R., Shivappa N., Wirth M.D., Hussey J.R., Hurley T.G. (2019). Perspective: The Dietary Inflammatory Index (DII)—Lessons Learned, Improvements Made, and Future Directions. Adv. Nutr..

[28] Shivappa N., Steck S.E., Hurley T.G., Hussey J.R., Hébert J.R. (2014). Designing and developing a literature-derived, population-based dietary inflammatory index. Public Health Nutr..

[29] Wirth M.D., Robinson C., Murphy E.A., Shivappa N., Hébert J.R. (2020). The dietary inflammatory index is associated with gastrointestinal infection symptoms in the national health and nutrition examination survey. Int. J. Food Sci. Nutr..

[30] McDiarmid K.P., Wood L.G., Upham J.W., MacDonald-Wicks L.K., Shivappa N., Hebert J.R., Scott H.A. (2022). The Impact of Meal Dietary Inflammatory Index on Exercise-Induced Changes in Airway Inflammation in Adults with Asthma. Nutrients.

[31] Vahid F., Rahmani D. (2021). Can an anti-inflammatory diet be effective in preventing or treating viral respiratory diseases? A systematic narrative review. Clin. Nutr. ESPEN.

[32] Sudlow C., Gallacher J., Allen N., Beral V., Burton P., Danesh J., Downey P., Elliott P., Green J., Landray M. (2015). UK Biobank: An Open Access Resource for Identifying the Causes of a Wide Range of Complex Diseases of Middle and Old Age. PLoS Med..

[33] Bradbury K.E., Young H.J., Guo W., Key T.J. (2018). Dietary assessment in UK Biobank: An evaluation of the performance of the touchscreen dietary questionnaire. J. Nutr. Sci..

[34] McCance R.A., Widdowson E.M. (2014). McCance and Widdowson’s the Composition of Foods.

[35] Liu B., Young H., Crowe F.L., Benson V.S., Spencer E.A., Key T.J., Appleby P.N., Beral V. (2011). Development and evaluation of the Oxford WebQ, a low-cost, web-based method for assessment of previous 24 h dietary intakes in large-scale prospective studies. Public Health Nutr..

[36] Townsend P., Phillimore P. (1988). Beattie: A Health and Deprivation: Inequalities and the North.

[37] SAS Institute Inc. (2017). The CAUSALMED Procedure. SAS/STAT 14.3 User’s Guide.

[38] Merino J., Joshi A.D., Nguyen L.H., Leeming E.R., Mazidi M., Drew D.A., Gibson R., Graham M.S., Lo C.-H., Capdevila J. (2021). Diet quality and risk and severity of COVID-19: A prospective cohort study. Gut.

[39] El Khoury C.N., Julien S.G. (2021). Inverse Association between the Mediterranean Diet and COVID-19 Risk in Lebanon: A Case-Control Study. Front. Nutr..

[40] Ponzo V., Pellegrini M., D’Eusebio C., Bioletto F., Goitre I., Buscemi S., Frea S., Ghigo E., Bo S. (2021). Mediterranean Diet and SARS-COV-2 Infection: Is There Any Association? A Proof-of-Concept Study. Nutrients.

[41] Menzel A., Samouda H., Dohet F., Loap S., Ellulu M.S., Bohn T. (2021). Common and Novel Markers for Measuring Inflammation and Oxidative Stress Ex Vivo in Research and Clinical Practice—Which to Use Regarding Disease Outcomes?. Antioxidants.

[42] Hebert J.R., Hebert J.R., Hofseth L.J. (2022). Chapter 2. History of nutrition and inflammation. Diet, Inflammation, and Health.

[43] Furuyashiki T., Akiyama S., Kitaoka S. (2019). Roles of multiple lipid mediators in stress and depression. Int. Immunol..

[44] Serhan C.N. (2014). Pro-resolving lipid mediators are leads for resolution physiology. Nature.

[45] Cecconello C., Ribas P.C., Norling L.V., Hebert J.R., Hofseth L.J. (2022). Chapter 4. Resolving acute inflammation; what happens when inflammation goes haywire? How can it get back in line?. Diet, Inflammation, and Health.

[46] Lee Y.S., Olefsky J. (2021). Chronic tissue inflammation and metabolic disease. Genes Dev..

[47] Hofseth L.J., Hebert J.R., Hebert J.R., Hofseth L.J. (2022). Chapter 3. Diet and acute and chronic, systemic, low-grade inflammation. Diet, Inflammation, and Health.

[48] Ouchi N., Parker J.L., Lugus J.J., Walsh K. (2011). Adipokines in inflammation and metabolic disease. Nat. Rev. Immunol..

[49] Moludi J., Qaisar S.A., Alizadeh M., Vayghan H.J., Naemi M., Rahimi A., Mousavi R. (2022). The relationship between Dietary Inflammatory Index and disease severity and inflammatory status: A case–control study of COVID-19 patients. Br. J. Nutr..

[50] Marx W., Veronese N., Kelly J.T., Smith L., Hockey M., Collins S., Trakman G.L., Hoare E., Teasdale S.B., Wade A. (2021). The Dietary Inflammatory Index and Human Health: An Umbrella Review of Meta-Analyses of Observational Studies. Adv. Nutr..

[51] Phillips C.M., Chen L.-W., Heude B., Bernard J.Y., Harvey N.C., Duijts L., Mensink-Bout S.M., Polanska K., Mancano G., Suderman M. (2019). Dietary Inflammatory Index and Non-Communicable Disease Risk: A Narrative Review. Nutrients.

[52] Malcomson F.C., Willis N.D., McCallum I., Xie L., Shivappa N., Wirth M.D., Hébert J.R., Kocaadam-Bozkurt B., Özturan-Sirin A., Kelly S.B. (2021). Diet-Associated Inflammation Modulates Inflammation and WNT Signaling in the Rectal Mucosa, and the Response to Supplementation with Dietary Fiber. Cancer Prev. Res. Phila..

[53] Li S.X., Hodge A.M., MacInnis R.J., Bassett J.K., Ueland P.M., Midttun Ø., Ulvik A., Rinaldi S., Meyer K., Navionis A.-S. (2021). Inflammation-Related Marker Profiling of Dietary Patterns and All-cause Mortality in the Melbourne Collaborative Cohort Study. J. Nutr..

[54] Holmes B., Dick K., Nelson M. (2008). A comparison of four dietary assessment methods in materially deprived households in England. Public Health Nutr..

