# Supplementary material for: Diet-Related Inflammation Is Associated with Worse COVID-19 Outcomes in the UK Biobank Cohort
Source: Nutrients. 2023 Feb 9;15(4):884. doi: 10.3390/nu15040884 (PMC9959636; doi:10.3390/nu15040884)

Diet-related inflammation is associated with worse COVID-19 outcomes in the UK Biobank Cohort

Zhao et al.

Supplemental materials

Supplemental table 1. Sensitivity analyses on associations between DII, E-DII, and COVID-19 incidence, severity, and death in the UK Biobank

Supplemental figure 1. Study selection procedure

Supplemental figure 2. Nonlinearity plots for associations between DII and COVID-19 incidence, severity, and death in the UK Biobank.

Supplemental figure 3. Stratified analyses of associations between DII and COVID-19 incidence, severity, and death in the UK Biobank. RR showed for per SD increase in the DII. Model adjusted for age, sex, ethnicity, deprivation index, smoking status, history of heart diseases, cancer, and type 2 diabetes, physical activity, sleep duration except for the stratified factors.

Supplemental figure 4. Stratified analyses of associations between E-DII and COVID-19 incidence, severity, and death in the UK Biobank. RR showed for per SD increase in the E-DII. Model adjusted for age, sex, ethnicity, deprivation index, smoking status, history of heart diseases, cancer, and type 2 diabetes, physical activity, sleep duration except for the stratified factors.

Supplemental table 1. Sensitivity analyses for associations between DII, E-DII, and COVID-19 incidence in the UK Biobank

	Quintile 1	Quintile 2	Quintile 3	Quintile 4	Quintile 5	P for trend
**DII**						
Among participants with at least one COVID-19 report (n=68,820)	1 (reference)	0.99 (0.93-1.04)	1.06 (1.00-1.12)	1.07 (1.01-1.13)	1.05 (1.00-1.11)	0.004
Any positive reports in incidence or severity or death (n=196,154)	1 (reference)	0.99 (0.94-1.06)	1.09 (1.03-1.16)	1.09 (1.03-1.15)	1.10 (1.04-1.17)	< 0.001
Excluded participants with missing values in physical activity (n=167013)	1 (reference)	1.01 (0.95-1.08)	1.09 (1.02-1.16)	1.09 (1.03-1.16)	1.11 (1.05-1.19)	< 0.001
**E-DII**						
Among participants with at least one COVID-19 report (n=68,820)	1 (reference)	1.04 (0.98-1.10)	1.02 (0.97-1.08)	1.12 (1.06-1.18)	1.14 (1.08-1.20)	< 0.001
Any positive reports in incidence or severity or death (n=196,154)	1 (reference)	1.06 (0.99-1.12)	1.04 (0.98-1.11)	1.15 (1.09-1.22)	1.17 (1.10-1.23)	< 0.001
Excluded participants with missing values in physical activity (n=167013)	1 (reference)	1.06 (0.99-1.13)	1.02 (0.96-1.09)	1.15 (1.08-1.23)	1.18 (1.11-1.26)	< 0.001

Model adjusted for age, sex, ethnicity, deprivation index, smoking status (except for non-smokers analyses), history of heart diseases, cancer, and type 2 diabetes, physical activity, sleep duration.

Supplemental figure 1. Study selection procedure

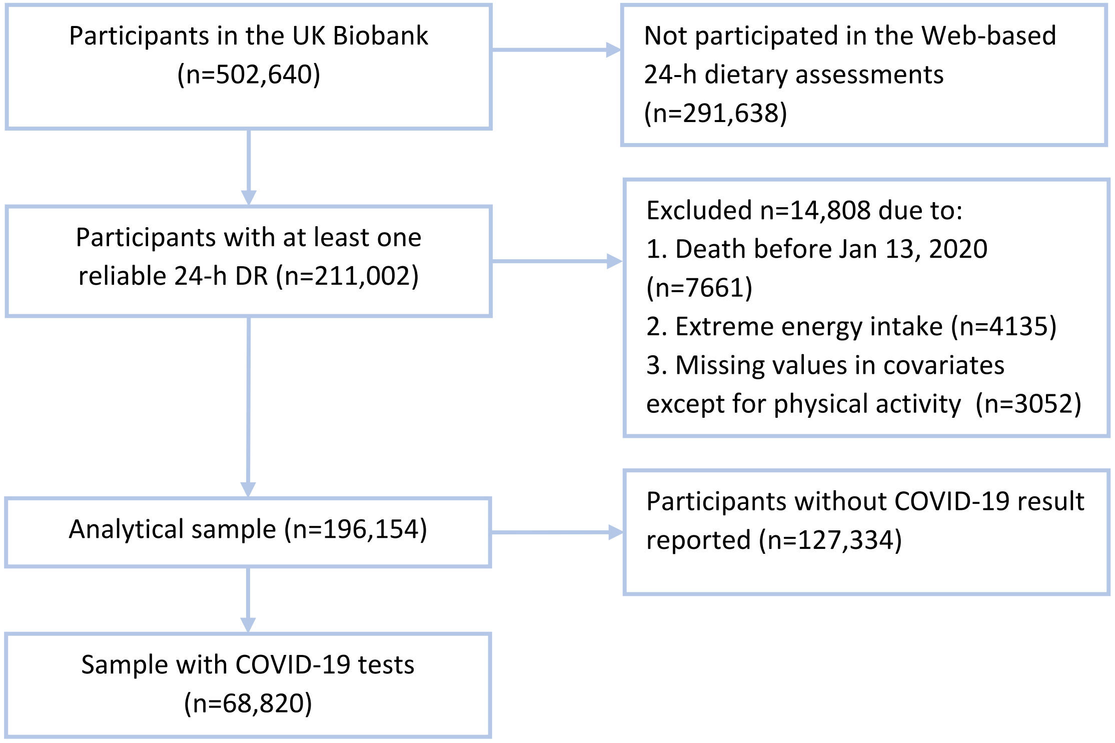

Supplemental figure 2. Nonlinearity plots for associations between DII/E-DII and COVID-19 incidence, severity, and death in the UK Biobank.

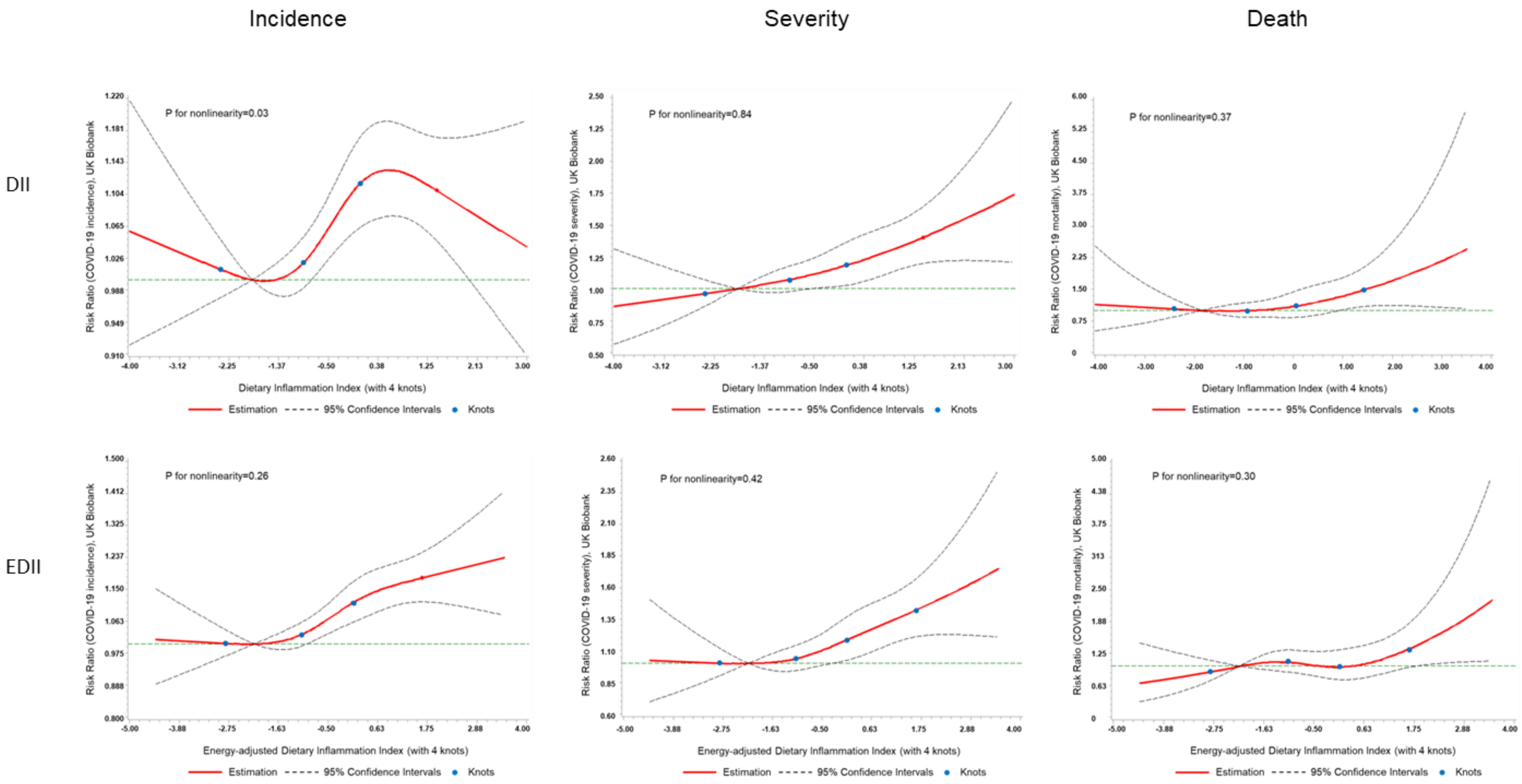

Supplemental figure 3. Stratified analyses of associations between DII and COVID-19 incidence, severity, and death in the UK Biobank. RR is presented per SD increase in DII. Model adjusted for age, sex, ethnicity, deprivation index, smoking status, history of heart diseases, cancer, and type 2 diabetes, physical activity, sleep duration except for the stratified factors.

	Incidence		Severity		Death	
	RR (95%CI)	$P_{interaction}$	RR (95%CI)	$P_{interaction}$	RR (95%CI)	$P_{interaction}$
**Sex**		0.98		0.36		0.33
Male						
Female						
**Ethnicity**		0.59		0.81		0.70
Non-Whites						
Whites						
**History of heart diseases**		0.38		0.49		0.31
No						
Yes						
**History of cancer**		0.64		0.98		0.97
No						
Yes						
**History of type 2 diabetes**		0.36		0.61		0.80
No						
Yes						
**Smoking status**		0.003		0.43		0.68
Never smoker						
Ever smoker						
**Sleep duration**		0.92		0.94		0.63
<7 hours						
≥7 hours						
**BMI category**		0.84		0.54		0.65
Normal weight						
Overweight and obese						

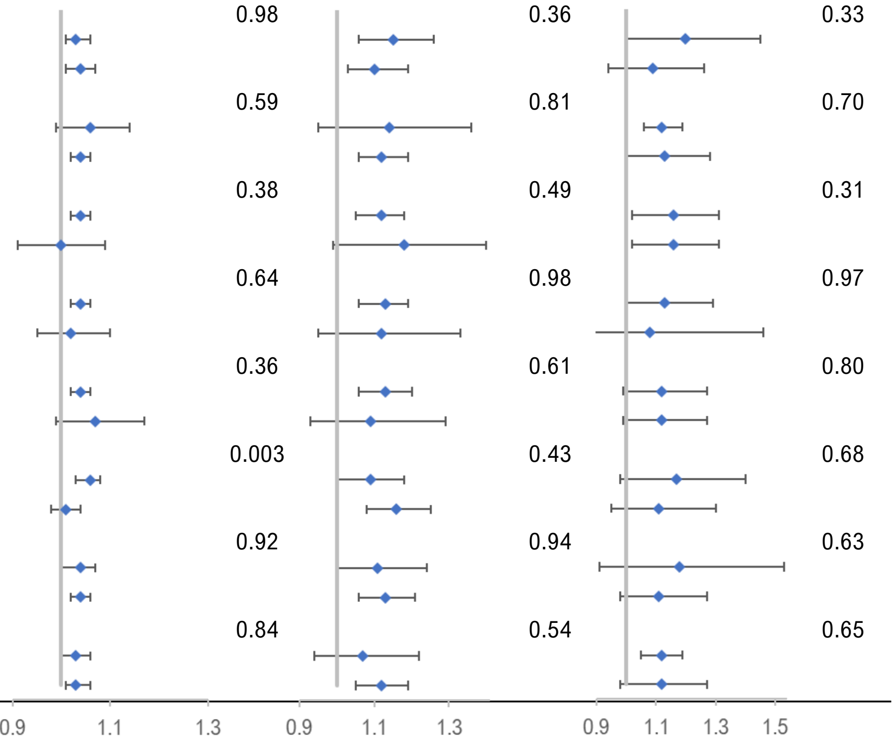

Supplemental figure 4. Stratified analyses of associations between E-DII and COVID-19 incidence, severity, and death in the UK Biobank. RR presented per SD increase in E-DII. Model adjusted for age, sex, ethnicity, deprivation index, smoking status, history of heart diseases, cancer, and type 2 diabetes, physical activity, sleep duration except for the stratified factors.

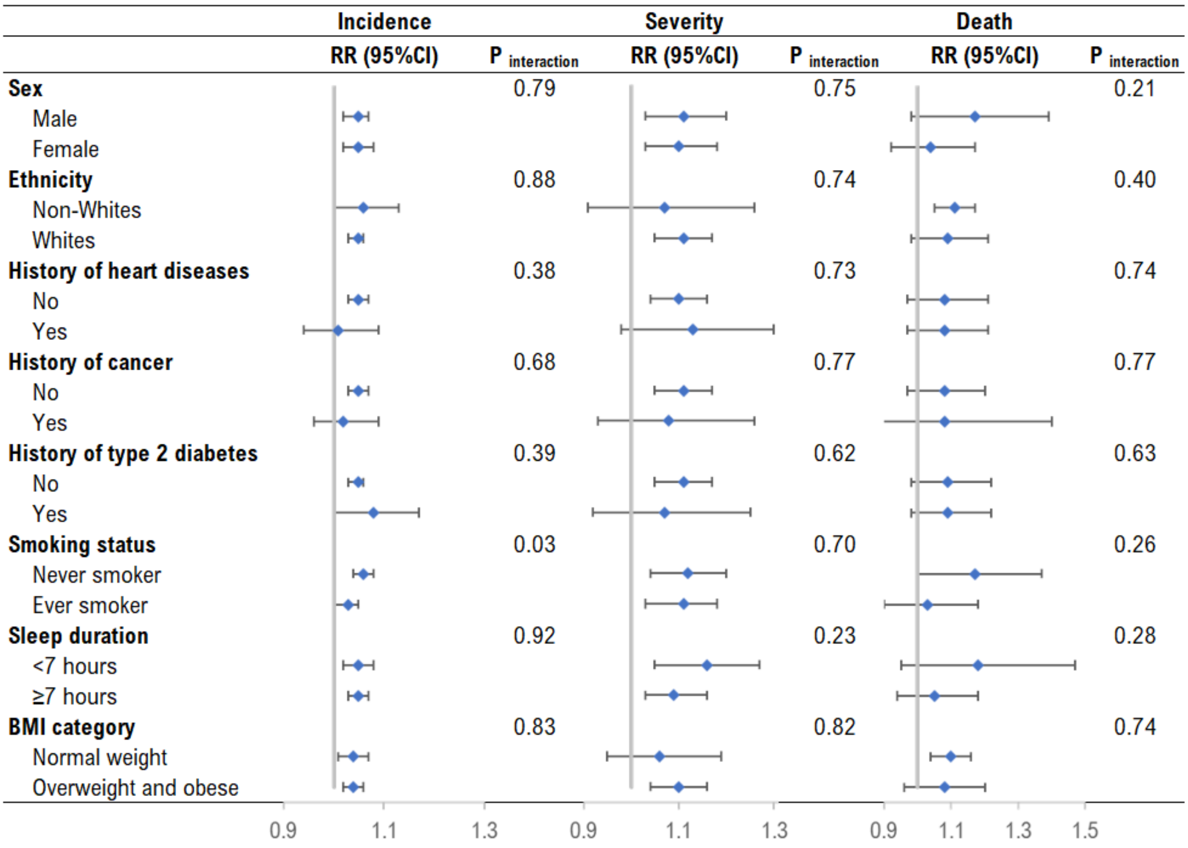

Supplement: Supplementary file 1 [file nutrients-15-00884-s001.zip › nutrients-2201555-supplementary.pdf]
